# Unraveling the Mysteries of Perineural Invasion in Benign and Malignant Conditions

**DOI:** 10.3390/curroncol30100647

**Published:** 2023-09-30

**Authors:** Hisham F. Bahmad, Samantha Gogola, Michael Rejzer, Kalin Stoyanov, Aaron S. Gomez, Ann-Katrin Valencia, Adonicah Cummings, Timothy Skerry, Ferial Alloush, Abed A. Aljamal, Arunima Deb, Sarah Alghamdi, Robert Poppiti

**Affiliations:** 1 The Arkadi M. Rywlin M.D. Department of Pathology and Laboratory Medicine, Mount Sinai Medical Center, Miami Beach, FL 33140, USA; ferial.alloush@msmc.com (F.A.); arunima.deb@msmc.com (A.D.); sarah.alghamdi@msmc.com (S.A.); robert.poppiti@msmc.com (R.P.); 2 Herbert Wertheim College of Medicine, Florida International University, Miami, FL 33199, USA; sgogo002@med.fiu.edu (S.G.); mrejz001@med.fiu.edu (M.R.); kstoy002@med.fiu.edu (K.S.); agome399@med.fiu.edu (A.S.G.); avale115@med.fiu.edu (A.-K.V.); acumm012@med.fiu.edu (A.C.); tsker001@med.fiu.edu (T.S.); 3 Department of Medicine, Division of Hematology Oncology, Medical University of South Carolina, Charleston, SC 29425, USA; aljamal@musc.edu; 4 Department of Pathology, Herbert Wertheim College of Medicine, Florida International University, Miami, FL 33199, USA

**Keywords:** perineural invasion, TNM classification, staging, College of American Pathologists, World Health Organization

## Abstract

Perineural invasion (PNI) is defined as the dissemination of neoplastic cells within the perineural space. PNI can be a strong indicator of malignancy and is linked to poor prognosis and adverse outcomes in various malignant neoplasms; nevertheless, it can also be seen in benign pathologic conditions. In this review article, we discuss various signaling pathways and neurotrophic factors implicated in the development and progression of PNI. We also describe the methodology, benefits, and limitations of different in vitro, ex vivo, and in vivo models of PNI. The spectrum of presentation for PNI can range from diffuse spread within large nerves (“named” nerves) all the way through localized spread into unnamed microscopic nerves. Therefore, the clinical significance of PNI is related to its extent rather than its mere presence or absence. In this article, we discuss the guidelines for the identification and quantification of PNI in different malignant neoplasms based on the College of American Pathologists (CAP) and World Health Organization (WHO) recommendations. We also describe benign pathologic conditions and neoplasms demonstrating PNI and potential mimics of PNI. Finally, we explore avenues for the future development of targeted therapy options via modulation of signaling pathways involved in PNI.

## 1. Introduction

**Figure 1:**
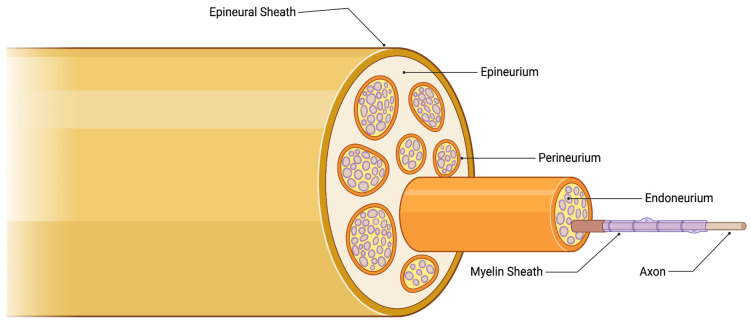
The anatomy of the nerve sheath. Created with BioRender.com (2023).

Perineural invasion (PNI) is defined as the dissemination of neoplastic cells within the perineural or intraneural space [1] (Figure 1). This allows the associated nerves to act as a pathway for metastasis, even in cases where blood or lymph metastasis is absent [2]. Tumor cells may be found in any layer of the nerve sheath; however, perineural involvement should also be suspected in tumors in close proximity to a nerve that is at least one-third the size of the nerve’s circumference [3]. Thus, this phenomenon can be classified into four subtypes based on the extent of involvement: tumor cells in proximity to nerves; tumor cells encircling nerves less than 33%; tumor cells encircling nerves greater than 33%; and tumor cells infiltrating the nerve sheath [4].

Effective methods of detection of PNI include histopathological morphology on hematoxylin and eosin (H&E) stain, S-100 protein immunohistochemical (IHC) staining with or without double staining for cytokeratin AE1/3, magnetic resonance (MRI), and positron emission tomography–computed tomography (PET-CT) [3,4,5]. Imaging techniques can also detect PNI in those who are symptomatic [5]. While MRI is the modality of choice, it is difficult to use when assessing the extent of disease due to the presence of microscopic tumor spread and skip lesions [5]. For these reasons, PET-CT may be used concomitantly to identify areas of increased metabolic activity [5].

PNI can be a strong indicator of malignancy and is associated with poor patient prognosis and low survival rates in certain tumors [2]. As such, it has been well documented in cases of prostate cancer, pancreatic ductal adenocarcinoma (PDAC), gastric cancer, colorectal cancer (CRC), gallbladder cancer, cervical cancer, and squamous cell carcinoma (SCC), among others [2].

## 2. Signaling Pathways Involved in Inducing Perineural Invasion

**Figure 2:**
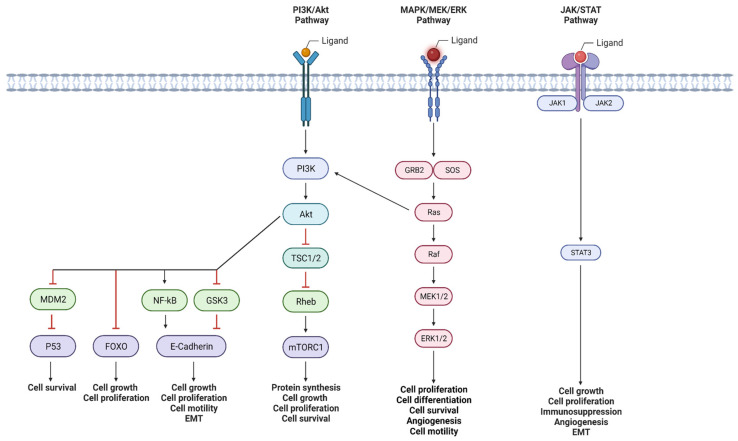
Outline three major cell signaling cascades implicated with the induction of perineural invasion. Created with BioRender.com (2023).

There are several signaling pathways implicated in the induction of PNI. The phosphoinositide 3-kinase (PI3K)/Akt pathway regulates vital cellular functions, and its hyperactivation, caused by certain genetic alterations, is linked to PNI in various cancers. Mitogen-activated protein kinase (MAPK) pathways, crucial for cell proliferation and differentiation, are also connected to PNI induction. Additionally, Janus kinase/signal transducer and activator of transcription (JAK/STAT) pathway modulation, known for immune regulation, influences PNI by promoting cancer survival and migration. Other pathways, like Sonic Hedgehog (SHH), transforming growth factor-β (TGF-β)/SMAD, and Wnt/β-catenin, among others, are being increasingly recognized for their roles in PNI initiation. These pathways’ roles make them targets for potentially inhibiting PNI, contributing to advancements in cancer research and treatment (Figure 2).

### 2.1. PI3K/Akt Pathway

The PI3K/Akt signaling pathway is involved with many important cellular processes, including proliferation, growth, metabolism, survival, and apoptosis [6]. In general, the pathway begins with the extracellular binding of a signaling ligand to a membrane-bound receptor tyrosine kinase (RTK), which results in autophosphorylation of the RTK and recruitment of PI3K [6]. This activation initiates a signaling cascade that includes an important family of serine/threonine kinases known as Akt [6]. The three isoforms of Akt act as a relay point for signal propagation to various downstream effector molecules, including, but not limited to, glycogen synthase kinase 3 (GSK3), which modulates cellular metabolism; forkhead box O (FOXO) transcription factors that regulate cell growth and proliferation; and mammalian target of rapamycin (mTOR) complexes involved with cell survival and apoptosis [7].

With its central role in cellular growth and survival, the PI3K/Akt pathway has been widely implicated in the progression of many human cancers [8]. Genetic alterations at each level of the signaling cascade have been shown to induce pathway hyperactivation that contributes to carcinogenesis, tumor growth, metastasis, and drug resistance [8]. As such, there has been significant interest in the potential of the PI3K/Akt pathway to induce PNI. Alkhadar et al. demonstrated a direct role of Akt activation in promoting tumor cell scattering and migration in several oral and salivary gland cancers [9]. Jiang et al. presented evidence of PI3K/Akt/GSK signaling to promote PNI in PDAC via enhanced proliferation and epithelial-to-mesenchymal transition (EMT) [10]. Wang et al. further supported a PI3K/Akt-mediated, EMT-based mechanism of PNI induction in their recent CRC investigation [11].

### 2.2. MAPK Pathway

MAPK signaling pathways play a critical role in the regulation of cell proliferation, differentiation, apoptosis, angiogenesis, and motility [12]. Similar to the PI3K/Akt pathway, signal induction generally begins with the extracellular binding of a signaling ligand to a membrane-bound RTK [12]. This results in autophosphorylation of the RTK and activation of associated proteins such as growth factor receptor-bound protein 2 (GRB2), son of sevenless (SOS), and the critical GTPase, Ras. Activation of Ras initiates a cascade of protein kinases that includes Raf, mitogen-activated protein kinase (MEK), and extracellular signal-regulated kinase (ERK) [12]. Activated ERKs translocate from the cytoplasm to the nucleus, where they regulate various transcription factors, including, but not limited to, ETs-like gene 1 (ELK-1), which promotes cell proliferation, c-Myc, which regulates cyclin-mediated cell cycle progression, and B-related factor 1 (BRF1), which activates ribosome biogenesis [13,14,15].

The established roles of MAPK signaling pathways in cellular proliferation and survival have made them popular targets for investigating tumor proliferation, anti-apoptotic survival, invasion and metastasis, and angiogenesis [16]. Furthermore, a growing body of research has begun to connect MAPK pathway activation with the induction and progression of PNI. Huang et al. identified that stimulation of the ERK pathway induced PDAC cell migration, invasion, and PNI through the promotion of epithelial-to-mesenchymal transition [17]. Veit et al. similarly explored the induction of PNI in PDAC, demonstrating a critical role of the Ras/Raf/MEK/ERK pathway in tumor cell migration and PNI via actin filament reorganization [18]. Beyond PDAC, Gao et al. showed that MAPK activation of the proto-oncogene *ETS1* induced PNI in CRC cells via the expression of matrix metalloproteinases (MMPs) [19]. This connection between MAPK activation, MMP expression, and PNI induction was further demonstrated in oral SCC samples by Chuang et al. [20].

### 2.3. JAK/STAT Pathway

The JAK/STAT pathway plays a central role in modulating the immune system, hematopoiesis, inflammation, apoptosis, and adipogenesis [21]. Signal transduction begins with a ligand binding to its cell surface, the transmembrane receptor, which induces a conformational change in the receptor that activates associated JAKs [21]. The activated JAKs subsequently recruit and activate various STATs, resulting in STAT nuclear translocation, promoter sequence binding, and transcriptional modulation of specific genes [21].

Members of the JAK/STAT pathway have a well-established role in tumor survival and progression, with STAT3 alone promoting cell cycle progression, elevated metabolism, immunosuppression, angiogenesis, and EMT in multiple cancers [22]. With this in mind, growing evidence has implicated JAK/STAT signaling in the induction of PNI. Guo et al. outlined a significant role for STAT3 in promoting PNI in PDAC cells through enhanced MMP activity [23]. Bressy et al. investigated PNI induction in PDAC, highlighting the involvement of JAK/STAT3 signaling in the induction of neuronal plasticity and Schwann cell migration [24]. Beyond PDAC, Zhang et al. highlighted an important role of JAK/STAT activation in gastric cancer cell migration and MMP-mediated invasion, both important aspects of PNI [25].

### 2.4. Other Pathways

Beyond the major pathways highlighted thus far, a growing number of additional signaling cascades have been implicated in the initiation and progression of PNI. Li et al. demonstrated that elevations in PDAC cell SHH signaling activated SHH signaling in stromal stellate cells, resulting in enhanced PNI through neuronal outgrowth to cancer colonies [26]. Qin et al. revealed an important role of TGF-β/SMAD signaling in PDAC PNI via the upregulation of EMT-associated genes and the promotion of neuronal axon growth [27]. Hassounah et al. investigated the Wnt/β-catenin pathway in prostate cancer and found an associated dysfunction of primary cilia that correlated with PNI foci [28].

## 3. Neurotrophic Factors and Their Functions and Expression Patterns in Cancer

**Figure 3:**
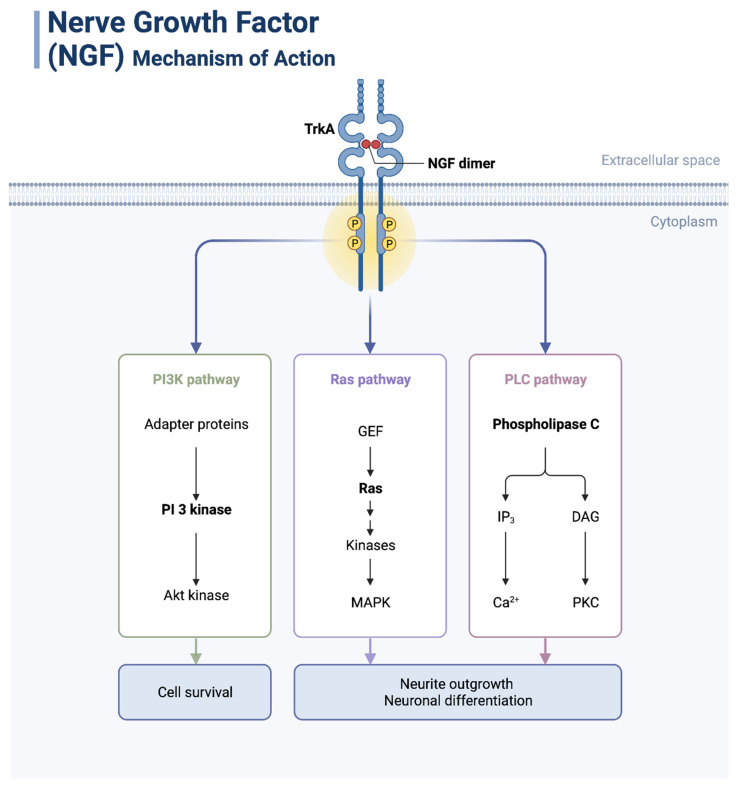
Nerve growth factor (NGF) mechanism of action. NGF dimer binds to its receptor TrkA to activate the PI3K, Ras, and PLC pathways to promote cell survival, neurite outgrowth, and neuronal differentiation. Created with BioRender.com (2023).

Neurotrophic factors have been implicated in the pathogenesis of PNI [29]. These protein molecules, generated by nerve tissue and astrocytes, are necessary for the growth and survival of nerve cells [2]. In tumor nerve cells, they play an important role in the regulation of several signal pathways and may facilitate neoplastic growth and directional dissemination of cancer along nerves [30] (Figure 3).

### 3.1. NGF

Nerve growth factor (NGF) is required for the survival of the sympathetic nervous system and sensory neurons [2,29]. The release of NGF by Schwann cells suggests that it is also involved in glial cell migration through PNI [29]. In cancer cells, NGF promotes neuritic growth, down-regulates apoptosis, and increases tumor proliferation [2,29,30]. One of its receptors, P75NTR, has been indicated as a chemoattractant for tumor cells toward neural tissue, thus promoting PNI [29]. High levels of NGF and TrkA, another of its receptors, have been correlated with increased frequency and severity of PNI and pain [29]. Exposure of PDAC cells to exogenous NGF resulted in increased MMP-2 expression, potentially accounting for increased invasiveness due to its role in the extracellular matrix [2,30].

### 3.2. GDNF

Glial cell line-derived neurotrophic factor (GDNF) has high expression in several types of tumors and is involved in many signaling pathways for growth and differentiation, the growth of nerve axons, and cell survival [2]. GDNF-RET association and the receptor GFRa1 are especially significant in PNI, with downstream activation of MAPK to enhance the invasion of cells and promote the formation of axons [2,29,30]. Via the JAK2/STAT1 pathway, GDNF can induce the expression of programmed death-ligand 1 (PD-L1), leading to immunosuppression [2]. Artemin, one of four GFNF proteins, and its receptor GFRa3 have also been implicated in the generation of PNI in PDAC and the promotion of neurotrophic invasion [2,30].

### 3.3. BDNF

Brain-derived neurotrophic factor (BDNF) encourages the survival of already existing neurons and promotes the development of new neurons and their synapses [30]. Its primary receptor is TrkB, with overexpression in several tumor cells that activate MMP-2 to cause increased invasiveness and poor prognosis [2,29,30]. Prior in vitro studies linked the overexpression of BDNF to increased proliferation and invasion of neoplastic cells; however, in vivo studies did not have the same outcome [29].

### 3.4. NT-3

Neurotrophin-3 (NT-3) supports the growth and differentiation of new and established neurons [30]. NT-3 and its receptor TrkC have been shown to be highly expressed in many different tumor types, especially PDAC [2,30]. In prior studies, blocking NT3 inhibited the survival and growth of prostatic adenocarcinoma and PDAC in a murine xenograft model, suggesting a role in invasion [30].

## 4. Experimental Models of Perineural Invasion in Cancer

The understanding of PNI as a neoplastic process has evolved, especially in the past century [31]. It was initially thought to be, broadly speaking, the invasion of tumor cells within the vicinity of peripheral nerves or within these nerves themselves, a definition first proposed by J. G. Batsakis in 1985 [32]. However, due to the lack of specificity in attempting to apply this definition of PNI, further elucidation was needed to correctly describe what was being observed in tissue samples [33]. This was a partial definition, and the need for greater clarity led to a further change in the description of PNI. PNI was then described as the presence of a tumor near a peripheral nerve that either envelopes 33% or more of the nerve’s circumference or has tumor cells present within the epineurium, perineurium, or endoneurium of neighboring nerves [34]. These defining features of PNI are currently widely acknowledged [35]. With the evolving perspective of PNI comes the need to represent this phenomenon in various biological models to further enhance our understanding of this seemingly unique avenue of pathogenesis, distinct from both lymphatic and vascular invasion [36]. Presently, in vitro, ex vivo, and in vivo models are being utilized to attempt to replicate PNI in humans [35] (Table 1).

When discussing in vitro models of PNI, the two most widely used are the Transwell and dorsal root ganglia co-culture models [35]. The Transwell model is a two-dimensional model that employs Transwell inserts [35]. Dorsal root ganglia that are harvested from either newborn rats or mice are placed in the basolateral chamber of the insert, and the cancer cells of interest are placed in the apical membrane of the insert, which is coated in Matrigel, a basement membrane matrix [35]. This particular model has primarily been used to study chemotactic mechanisms involving cytokines released by nerves or chemokines that are present on the cell surface of the cancer of interest [35]. While the Transwell model is straightforward, easy to replicate, and a cost-effective means of studying PNI, it is limited in its ability to accurately reproduce the full range of behavior in the extracellular domain observed by cancer cells [35]. The dorsal root ganglia co-culture model refers to the employment of a three-dimensional culture method wherein dorsal root ganglia are harvested from either newborn rats or mice and inserted into pre-treated Matrigel contained within a transparent culture plate [37]. The inserted dorsal root ganglia are subsequently given adequate time to culture and begin to develop axonal projections in a radial fashion, which typically occurs within a span of seven days [37]. Cancer cells of interest are fluorescently tagged and then inserted within the same Matrigel matrix in the vicinity of the cultured dorsal root ganglia [37]. The proximity of the neural and cancer cells allows for invasion to be observed as fluorescence within the neural cells [35]. The employment of a transparent culture plate allows for further analysis to be made with techniques such as time-lapse microscopy, allowing for enhanced visualization of cellular movement within the extracellular milieu as well as intercellular interactions [37]. This second in vitro PNI model also allows for samples to be collected from the culture itself to be analyzed from a molecular standpoint [35].

Two models that have been developed to study PNI ex vivo are the explanted nerve and organoid models [35]. Abiatari et al. established the explanted nerve model through the use of vagus nerves harvested from rats [38]. The harvested vagus nerves were placed at the base of a chamber of unique design that possessed a gap of 0.7 mm [38]. In order to observe nerve invasion with this method, the chamber was placed on a standard culture medium [38]. Cancer cells of interest were then inserted into the chamber, allowing for the potential for said cells to invade the vagus nerves and reach the standard culture medium below [38]. The presence or absence of cancer cells in the medium of the culture plate in this model provides researchers with a valuable means of determining the potential of neural invasion [35]. The explant model was elaborated through the use of sciatic nerves harvested from mice [39]. In this particular variant of the model, cancer cells were propagated in 24-well plates within which the harvested sciatic nerves would be placed [39]. After a 48-h period, the sciatic nerves were removed and processed into a paraffin block for subsequent histologic analyses [39]. The sciatic nerve variant of the explanted nerve model has allowed for analysis of the impact of tumor suppressors on PNI, axonal proliferation, and the interplay between cancer cells of interest and Schwann cells [35]. The second ex vivo model used to study PNI, the organoid model, involves the co-culturing of dorsal root ganglia from young mice with organoids representative of neoplasms, such as pancreatic intraepithelial neoplasms or murine pancreatic organoids, via a Transwell insert [40]. The use of this system has allowed for further investigation of intercellular signaling between cancer cells and neurons while also accurately replicating the neural microenvironment [35].

Several models have been developed over time to study PNI in vivo, as this has the potential for optimal replication [35]. The first category of in vivo models is referred to as the heterotopic xenograft model [35]. The first specific model in this category that has seen widespread use utilizes murine sciatic nerves [41]. These sciatic nerves have cancer cells inserted into the neural sheath, specifically the perineurium, and subsequently observed for evidence of proliferation and invasion, such as keeping track of tumor growth or the preservation of the sciatic nerve’s function [42]. This methodology can measure tumor growth via conventional radiologic imaging, such as magnetic resonance techniques [35]. In addition, the tissues of interest can be harvested and undergo histologic and chemical analyses when needed [35]. The second type of xenograft model involves the insertion of cancer cells subcutaneously in nude mice and is later analyzed histologically for PNI frequency [43]. In an effort to most accurately replicate PNI in humans, the orthotopic xenograft model has seen implementation [35]. This particular model requires that cancer cells be directly inserted into the organs of the studied mice, such as the prostate, which are later harvested and analyzed for PNI frequency [44]. While heterotopic xenograft models provide an avenue by which the molecular aspects of PNI can be investigated, they are limited by their inability to display concurrent neural alterations that would be seen in humans [35]. Genetically engineered mouse models have also seen implementation in efforts to study PNI [35]. The current understanding of cancer genetics and carcinogenesis has allowed for the genetic manipulation of mice, resulting in highly accurate replications of human cancers for research purposes [45].

While this may seem promising as a tool for PNI research at face value, the application of these models to study PNI has been limited. The neuroplasticity observed in these models is restricted compared to what is seen in humans, and there appears to be a lack of significant neural invasion observed in the genetically engineered mouse models [46]. The last of the in vivo models used to investigate PNI is the chicken embryo, chorioallantoic membrane (CAM)-dorsal root ganglia model [47]. This final model takes advantage of the fact that the chorionic epithelium and human epithelium bear a striking similarity in their composition, with both possessing a basement membrane that has large amounts of type IV collagen [48]. The CAM-dorsal root ganglia model in this context was initially used to investigate basement membrane invasion by cancer cells. Later, it was repurposed as a means of studying PNI by grafting dorsal root ganglia harvested from Sprague–Dawley rats into the chorionic epithelium [49]. By later inserting cancer cells within the vicinity of the grafted dorsal root ganglia, the model aims to mimic a microenvironment of neural invasion [49]. While this model has allowed for the analysis of the roles that particular molecules or signaling pathways play in PNI, it is limited due to the inherently restricted observation period as a result of embryologic immune activation [35].

## 5. Clinical Significance of Perineural Invasion in Cancer

Although PNI has been identified as an important modality of tumor spread for over a century, it has historically been poorly understood. PNI describes a method by which tumor cells can spread, both within nerve fascicles and around nerve sheaths. The extent of invasion and spread can be highly variable, with different tumors and subtypes exhibiting different behaviors. The spectrum of presentation for PNI can range from diffuse spread within large nerves (“named” nerves), visualized through magnetic resonance imaging (MRI), all the way through localized spread into unnamed microscopic nerves.

Previous understanding of PNI was thought to be heavily related to the tumor attempting to find a “low-resistance” path of invasion; however, developments in the literature show that our understanding of PNI has changed. Quite the opposite effect was found, with perineural routes of invasion frequently having higher resistance due to multiple layers of intertwining collagen and basement membranes, as well as occluding junctions, causing greater cellular adhesion and a highly impermeable and selective barrier [31]. IHC staining has been used to uncover that proteinases (MMP-2 and MMP-9 in particular) are utilized to break through the rigid extracellular matrix previously mentioned, providing a new avenue for tumor cells to invade [31,50]. While research into PNI is ongoing, its pathophysiology is currently believed to be a complex, multi-step process in which particular tumor microenvironments are favorable toward axonogenesis [51]. To briefly illustrate this dynamic, a currently proposed mechanism suggests a reciprocal relay between the native neural tissue and the tumor attempting to invade. The tumor secretes local growth factors that stimulate neural remodeling and growth within neural tissue, and native tissue responds to the input through accelerated axonogenesis and neurite formation [52]. These characteristics of the tumor continue in an uncontrolled fashion, as is typical for cancer, which allows the tumor to crawl forward, using the existing neural pathways as a scaffold for invasion. It is important to note that there are several proposed mechanisms for PNI, and a collective understanding of pathophysiology is developing.

### 5.1. Perineural Invasion as a Diagnostic Marker of Malignancy in Certain Tumors

While PNI may be commonly found in many cancers (and their respective subtypes), there are several tumors in which PNI is statistically found at a greater frequency. These tumors will be discussed in several successive passages. Additionally, PNI can be considered an identifiable feature in several cancers. Since PNI describes and signifies invasion, it can generally be considered a diagnostic marker of malignancy. However, it is important to note that this is a generalization, and considerable variability in clinical outcomes exists between the different tumors found to have features of PNI.

#### 5.1.1. Prostate Cancer

It has been suggested that PNI is an identifiable histological feature in prostate cancer in up to 84% of cases [31,53]. While PNI is frequently encountered in prostate cancer, its significance remains controversial. In terms of prognosis, few studies have shown that PNI is associated with advanced prostate cancer and an increased risk of biochemical recurrence, even after prostatectomy or radiotherapy [54,55]. While PNI could, in reality, serve as an independent prognostic factor for patients with prostate cancer, a major caveat exists. There is no consensus on the significance of PNI concerning the prognosis, treatment, or recurrence rates of prostate cancer. The perplexities associated with the diagnosis of PNI persist throughout the literature, and the attainment of conclusive results is hampered by multiple contributing factors. These factors include limitations in study designs, historically varying definitions of what PNI exactly is, and how different patient groups are stratified and analyzed within the respective studies [54,56]. For example, Zareba et al. remarked that in their 2017 study, lower rates of PNI (averaging 44%) were found in prostate samples compared to previous studies (which ranged between 50% and 80% prevalence of PNI). Zareba et al. stated that this was likely due to the usage of a strict criterion for PNI and the lack of ambiguity in defining PNI within the 2017 study [54].

Since studies have shown conflicting results (primarily due to variations in study design, patient populations, treatment status, and differing definitions of PNI across studies) regarding its impact on prognosis and treatment outcomes, further research is needed on the topic for greater clarity on whether PNI changes treatment modalities for prostate cancer. For example, Merrilees et al. and Ng et al. found that PNI has no prognostic significance for recurrence rates in patients who have undergone radical prostatectomy [57,58]. This result shows how specified the research is in terms of the significance of PNI. The study by Merrilees et al. explicitly assessed recurrence rates in post-prostatectomy patients using a 2007 definition of PNI [57]. Since then, different researchers have used different definitions of PNI and found different results. It is crucial to ponder beyond a dichotomous “yes/no” categorization for PNI, as recent research has found that calculating involvement in percentages rather than the previously mentioned dichotomous presence of PNI has a higher prognostic value [59]. Quantifying the percentage of nerves in which the tumor has invaded or is involved displays a far fuller view of the characteristics of the disease and its progression in the specific patient.

#### 5.1.2. Skin Cancers

While PNI is uncommon for some skin cancers, there are certain types, such as desmoplastic melanomas or SCC, where PNI can alarmingly change the prognosis and has been associated with treatment resistance and worse outcomes [60]. In the context of these specific tumors, the discovery of PNI can lead the clinician to consider radiotherapy due to the worsened outcomes, difficulty of treatment, and increased rates of recurrence that these tumors possess [60]. As with all therapies, specific considerations for each patient are necessary, as are the specific areas of invasion for that cancer. For example, PNI in cranial nerves tracking back into the skull would carry obvious risks with radiation treatment that would not be as substantial when compared to choosing to radiate an area of PNI within a distal nerve of a digit [61].

#### 5.1.3. Gastric Cancer

PNI has been found to be an independent prognostic factor that heightens the level of treatment recurrence rates and worsens both overall and disease-free survival in cases of gastric cancer [62,63]. PNI-positive gastric cancer is quite common, with reports between 30% and 70% of tumors showing histologic evidence of PNI. Clinically, treatment modalities have sometimes been divided into prognostic groups split between PNI-positive and PNI-negative. This fact illustrates the clinical significance of PNI [62,63].

#### 5.1.4. Pancreatic Ductal Adenocarcinoma

PDAC is a cancer in which rates of PNI approach nearly 100%, and the degree of PNI is a vital prognostic factor as to the clinical course of the tumor [64]. In fact, PNI is so frequent in PDAC that this cancer has its own sub-classification system to classify the location of PNI and guide the extent of resection needed for treatment (namely: extrapancreatic, intrapancreatic, intratumoral, and extratumoral PNI) [65]. It is important to note that PDAC may have many of these features when considering the degree of malignancy or characteristics of the tumor that are unique to a specific patient. To simplify, we will consider a comparison between patients with only extratumoral PNI and those with only intratumoral PNI. No significant difference in either overall survival or disease-free survival was observed when directly comparing these two groups, even though either one of these features provides a worse prognosis independently [66,67,68].

When discussing the intrapancreatic PNI variant, it is essential to note that it has notably lower survival rates compared to PDAC cases without PNI [69]. Despite curative resections, the intrapancreatic PNI variant has a median survival of 15 months compared to a median survival of 38.9 months for PDAC without intrapancreatic PNI [69]. Intrapancreatic PNI is also uniquely associated with increased tumor recurrence, specifically a 2.7 times greater risk compared to PNI-negative tumors [70]. Extrapancreatic PNI has also been found to significantly lower survival, even when found in lesions as small as 2.5 mm [67,68]. The 7th edition of the American Joint Committee on Cancer (AJCC) defines a <2 cm tumor as a T1 stage, which may be considered an early tumor. Following this rationale, extrapancreatic PNI is considered a poor prognosticator of disease progression even when detected at sizes as small as 2.5 mm, illustrating how aggressive this subtype can be [68,71].

PDAC is notoriously aggressive in its clinical course, and while the degree of PNI is closely related to outcomes, treatments are constrained mainly to surgical excision [64]. It is theorized that the reason for the high degree of PNI in this specific tumor is due to the anatomical location of the pancreas [64,72]. The pancreas is near the celiac and superior mesenteric plexuses, which regulate intricate neurophysiological functions for the nervous and digestive systems [72]. The pancreas does not directly touch these plexuses, which ordinarily provide input and output flows for the pancreas, aiding in its endocrine and exocrine functions. These convenient pathways allow the tumor to spread and invade [72].

#### 5.1.5. Gallbladder Cancer

The prevalence of PNI in gallbladder cancer has been found to be around 70% in advanced cases when assessing large-scale studies [73,74]. PNI is noted as a poor prognostic factor in gallbladder cancer, namely worsening survival in the context of remnant disease [73,75]. PNI status does not generally modify treatment courses in terms of resection compared to systemic treatment [75].

#### 5.1.6. Colorectal Cancer

The prevalence of PNI in CRC is roughly around 20% in large-scale studies [76]. PNI is considered to be an independent poor prognostic factor in CRC patients [76,77]. PNI-positive findings may worsen the prognosis of a stage II CRC with PNI, almost making it equally dangerous to the level of a stage III CRC without PNI [77].

#### 5.1.7. Other Tumors

Other tumors where PNI is seen include adenoid cystic carcinoma, with PNI being prevalent in around 50% of tumors [78]. It is also less commonly seen in papillary thyroid carcinoma, with PNI being prevalent in around 2% of cases [79], and invasive breast carcinoma, with PNI being prevalent in around 1% of cases [80].

### 5.2. Perineural Invasion as an Independent Predictor of Poor Prognosis in Certain Cancers

There are certain cancers in which PNI is considered to be an independent predictor of poor prognosis, further illustrating the importance of proper and timely identification of PNI by pathologists. Several tumors have been known to have significantly worse outcomes if PNI is found, including cutaneous SCC [60,81], CRC [82], gastric cancer [62], and oral SCC [83]. Treatment strategies are frequently modified when PNI is detected, as it worsens outcomes in these selected tumors.

#### 5.2.1. Cutaneous Squamous Cell Carcinoma

Within cutaneous SCC, the presence of PNI has been found within systematic reviews to be closely linked with poorer patient outcomes, specifically having been found to have a hazard ratio of 1.61 (1.24–2.09, 95% confidence interval) [81]. Consistent and proper identification of PNI for cutaneous SCC is important due to the sheer quantity of diagnoses since cutaneous SCC is the second most common skin carcinoma. While cutaneous SCC is considered to be on the side of easily treatable tumors in the majority of cases (95% of tumors can be curably excised), rates of recurrence can remain high [81,84]. PNI has been found to increase recurrence risks and is considered to be a high-risk, independent predictor of poor prognosis in cutaneous SCC [81,84].

#### 5.2.2. Colorectal Cancer

Within CRC, the presence of PNI has been found to significantly worsen progression, remission, and survivability [82]. Specifically, it has been reported that the presence of PNI in CRC has a hazard ratio of 2.17 (1.16–4.04, 95% confidence interval) in regard to CRC recurrence risks [82]. The hazard ratio of dying due to CRC is 2.12 (1.09–4.14, 95% confidence interval) when PNI is found [82].

#### 5.2.3. Gastric Cancer

Within gastric cancer, a systematic review and meta-analysis found that PNI significantly had a negative impact on overall survival and disease-free survival [62]. For overall survival, the hazard ratio was found to be 1.484 (1.237–1.781, 95% confidence interval) [62]. The disease-free survival hazard ratio was found to be 1.371 (1.230–1.527, 95% confidence interval) [62].

#### 5.2.4. Oral Squamous Cell Carcinoma

Within oral SCC, reports indicate that the presence of PNI has been found to have significant negative impacts on overall survival and disease-free survival rates [83]. PNI presence alters overall survival with a hazard ratio of 1.7 (1.40–2.22, 95% confidence interval) and disease-free survival with a hazard ratio of 1.84 (1.50–2.27, 95% confidence interval) [83].

### 5.3. Incorporation of Perineural Invasion Reporting in CAP Protocols of Some Tumors

The College of American Pathologists (CAP) has released several protocols for guiding the reporting and characterization of PNI in several tumors. PNI presence in biopsy or excision/resection samples may be significantly useful in risk stratification. This section includes examples incorporating the most recently released protocols as of July 2023, in which CAP found it appropriate (but not mandatory) to include PNI as a standard in reporting.

#### 5.3.1. Cutaneous Squamous Cell Carcinoma

In cutaneous SCC, PNI is considered by CAP to be a high-risk feature for primary tumor staging. Several criteria exist in this particular example, including PNI greater than 0.1 mm, PNI found deeper than the dermis, or evidence of involvement of named nerves [85].

#### 5.3.2. Prostate Cancer

In prostate cancer, the 2021 CAP protocols for case and specimen level reporting in the examination of prostate needle biopsies as well as resections state that PNI has been associated with extraprostatic extension (EPE), but as mentioned earlier in this review, PNI significance as a predictor of outcome and staging remains controversial [86]. Regardless, PNI is listed as a reportable feature in the CAP reporting templates (reporting is yet optional) [86].

#### 5.3.3. Gastric Cancer

In gastric cancer, the 2023 CAP reporting protocol contains a category on whether or not PNI is identifiable in the specimen (reporting it is optional) [87]. The protocol states that PNI has been shown to be an adverse prognostic factor and has associations with metastasis to lymph nodes in early gastric tumors [87].

#### 5.3.4. Colorectal Cancer

In CRC, the 2021 CAP protocol for examination of excisional biopsy specimens finds that PNI has been shown to be an independent predictor of poor prognosis, having a negative impact on survival in stage II CRC [88]. Within the reporting template on the document, PNI is not explicitly listed as a category; however, it is mentioned in the notes [88]. It is grouped together with “lymphovascular invasion” and is significant enough for CAP to mention this explicitly in this protocol [88].

#### 5.3.5. Gallbladder Cancer

In gallbladder cancer, the 2021 CAP reporting protocol contains a category on whether or not PNI is identifiable in the specimen (reporting it is optional) [89]. Within the notes of this guideline, CAP states that PNI is very common but is not always classified as an adverse prognostic factor in all studies [89]. CAP also explicitly notes that it is important to consider adenomyomatous hyperplasia as a differential when assessing gallbladder cancer pathology due to the involvement of perineural spaces [89].

#### 5.3.6. Pancreatic Ductal Adenocarcinoma

In PDAC, the 2021 CAP reporting protocol contains a category on whether or not PNI is identifiable in the specimen (reporting it is optional) [90]. Within the notes, it is plainly stated that PNI is an adverse prognostic factor [90]. There was no mention of conflicts in the literature regarding this worsened prognosis [90]. This is somewhat unique compared to some of the other previously listed guidelines, which mention that there are differences in significance and clinical outcomes of PNI-associated tumors that were found within the literature.

### 5.4. Incorporation of Perineural Invasion Reporting in WHO Protocols of Some Tumors

The following information is specific to the published works by the World Health Organization (WHO). The details regarding PNI in the discussed tumors are derived from the most recent versions of the “WHO Classification of Tumors” online series by the WHO Evidence Synthesis and Classification Branch. This section details the aforementioned tumors and what the WHO has found to be relevant in specimen reporting, staging, and clinical considerations.

#### 5.4.1. Cutaneous Squamous Cell Carcinoma

In cutaneous SCC, the “WHO Classification of Tumors” notes that PNI is relevant to the staging criteria if there is a primary tumor invasion greater than a diameter of 0.1 mm [91]. They also comment on the prognosis associated with PNI, stating that PNI greater than a diameter of 0.1 mm is associated with higher disease-specific death rates [91].

#### 5.4.2. Laryngeal Squamous Cell Carcinoma

In laryngeal SCC, the WHO notes different reporting criteria and features compared to cutaneous SCC. The “WHO Classification of Tumors” makes an explicit specification that PNI alters outcomes in laryngeal cancer due to the physics of tumor spread, as PNI is a preferential axis of spread in laryngeal SCC [92]. Contrastingly, the WHO finds insufficient evidence to include PNI as a parameter in pathology reporting datasets for these tumors [92].

#### 5.4.3. Prostate Cancer

In prostate cancer, particularly prostatic acinar adenocarcinoma, the referenced text, the “WHO Classification of Tumors,” explicitly notes that PNI is pathognomonic for prostate cancer [93]. It also notes that for PNI to be utilized as diagnostic criteria, the atypical prostate glands must circumferentially lap around the nerve or demonstrate invasion [93]. It is noted that sometimes benign prostate glands may groove and physically push the nerve [93]. PNI is also listed within the prostatic acinar adenocarcinoma diagnostic criteria as a “desirable” feature [93]. It is important to note that desirable in this context means that PNI is a desirable finding in establishing the diagnosis, not that PNI is desirable in the clinical setting. More information is listed on PNI within the context of this tumor compared to some of the other previously listed tumors. This may likely be due to prostate cancer being the most common malignancy found in men globally and acinar adenocarcinoma being the most frequent subtype [94]. These factors may have garnered greater attention for this particular tumor, as its prevalence cannot be overstated [94].

#### 5.4.4. Colorectal Cancer

In the CRC, the “WHO Classification of Tumors” notes that PNI is linked to increased rates of both local and distant recurrence, as well as decreased rates of survival [95]. This WHO report shows that PNI within CRC has been associated with a hazard ratio of 2.09 (1.68–2.61, 95% confidence interval) in univariate studies and a hazard ratio of 1.85 (1.63–2.12, 95% confidence interval) in multivariate studies when compared to CRC without PNI data, which were not explicitly provided for some previously discussed tumors [95]. These results speak for themselves and reaffirm the importance of identifying PNI in CRC. PNI is listed as a reportable criterion, indicating whether it is present or absent [95]. However, there is no explicit discussion on whether or not PNI is essential or if it is merely a desirable feature that helps guide the diagnosis of CRC.

#### 5.4.5. Pancreatic Ductal Adenocarcinoma

In PDAC, the “WHO Classification of Tumors” notes that PNI is a common feature due to the pancreas’ location near a copious quantity of nerves [96]. To illustrate the importance of this classic PDAC characteristic, the authors have listed the different histological criteria in the differential diagnosis of PDAC versus chronic pancreatitis, where PDAC is listed as perineural. This feature is useful in selecting PDAC as the proper diagnosis if PNI is present [96]. The text also mentions that PNI is a prognostic indicator in this tumor [96]. PNI is also explicitly listed as a “desirable” diagnostic criterion within the “Essential and desirable diagnostic criteria” section of the text [96].

## 6. Benign Neoplasms Demonstrating Perineural Invasion

Several benign cutaneous neoplasms demonstrate PNI, including congenital melanocytic nevus, blue nevus, granular cell tumor, infiltrating syringomatous adenoma, trichofolliculoma, and epithelial sheath neuroma. The pathogenesis of each benign cutaneous neoplasm is not well documented. However, there have been several well-studied mechanisms and hypotheses that explain the potential pathogenesis of PNI. The most validated hypothesis has been the interaction between tumor cells and nerves via chemical factors, signaling molecules, and neuropeptides. Part of these neuropeptides includes neurotrophic factors such as BDNF, NGF, NT-3, NT-4, the neural cell adhesion molecule (NCAM), chemokines, and galanin. Furthermore, these neurotrophic factors bind to receptors that induce signals to activate nuclear transcription factors that enhance neural cell growth and PNI. However, it would be essential to know which specific factors each distinct tumor secretes and their degree of involvement in the PNI mechanisms [97]. Ronaghy et al. reported various cases of benign cutaneous and non-cutaneous neoplasms, highlighting that PNI alone should not be used to classify a tumor as malignant. Certain features and criteria should be used to differentiate benign from malignant neoplasms to prevent unnecessary and potentially harmful treatment [98].

### 6.1. Congenital Melanocytic Nevus

Congenital melanocytic nevi (CMN) are congenital skin lesions that are present at or soon after birth and are derived from neural crest cells [99]. The most common clinical presentations for CMN are brown macules or papules that may be heterogeneous and most commonly present on the trunk. While estimates vary, there is a possible increase in the risk of malignancy with an increase in the size of the lesion [99,100]. Very few cases were found that directly reported PNI by CMN [101,102]. However, reviews by Alikhan et al. and Ronaghy et al. discussed how PNI by CMN may be more common than reported, with no indication that PNI is a sign of malignancy [98,99].

### 6.2. Blue Nevus

The blue nevus is a benign melanocytic lesion that most commonly presents as a cutaneous macule/papule with a blue color, with a predominance in the extremities, but it can also be found in the prostate, endometrium, and other tissues [103,104]. It has several clinical variants and histological variants. A review of the literature shows rare cases of PNI in blue nevi [98,105,106,107,108]. A case report from Min Young Lee showed a congenital plaque-type blue nevus that had PNI with no atypical markers present [109]. However, there is no indication in the literature that PNI in blue nevi is a sign of malignancy. Since blue nevi are benign in nature and can show PNI, it is important to identify them to prevent potential misdiagnosis of malignancy.

### 6.3. Granular Cell Tumor

A granular cell tumor (GCT) is a rare cutaneous benign lesion that originates from Schwann cells. It most commonly presents in the head/neck area, in particular the oral cavity, but can occur at any anatomic location [110,111]. It presents as a painless nodule in the dermis or subcutis tissue. Mobarki et al. described a review of 42 GCTs, 6 of which demonstrated PNI [112]. Stemm et al. conducted a study of 50 GCT, 4 of which demonstrated PNI [113]. Chow et al. described seven cases of GCTs forming within a nerve, as opposed to the typical extra-neural presentation [114]. Given their derivation from Schwann cells, it is unclear if PNI by GCTs is due to the inward invasion of the nerve or the tumor’s extension out of the nerve from its origin. However, PNI by GCTs does not show any increased risk for malignancy.

### 6.4. Infiltrating Syringomatous Adenoma

Infiltrating syringomatous adenomas (ISAs) are rare benign cutaneous lesions of the breast that are believed to arise from sweat ducts [115]. The most common presentation is in the subareolar region of the unilateral breast [116,117]. As indicated in the name, ISAs have an infiltrating histologic pattern and have been found to invade the perineural region. Jones et al. described 11 cases of ISA, only one of which demonstrated PNI [116]. Favre et al. described a case report of a 40-year-old woman with an ISA that was excised and examined to find PNI [115]. Both cases showed no evidence of further recurrence or malignancy. Although there is PNI in ISAs, the lack of distant metastases indicates it does not have the capability to become malignant.

### 6.5. Trichofolliculoma

Trichofolliculomas are very rare benign cutaneous tumors that originate from hair follicles. They most commonly present as a nodule on the head or face with protruding hair [118]. A review of the literature showed only one report of PNI by Trichofolliculomas by Stern and Stout [119]. No indication of malignancy was found, but long-term follow-up was recommended to determine recurrence and malignancy risk [98].

### 6.6. Epithelial Sheath Neuroma

An epithelial sheath neuroma (ESN) is a rare cutaneous benign neoplasm that is composed of a bundle of nerves wrapped in a sheath of epithelial cells [120]. The origin of ESNs is uncertain and has been postulated to be benign hyperplasia or hamartoma [121]. It is typically cured with excision and is not found to be aggressive [98]. Very few cases have been reported in the literature of ESN, and they have been shown to invade the perineural space [120,122,123,124]. It is important to be aware of the possibility of ESNs and their potential to show PNI in order to prevent the misdiagnosis of malignancy.

### 6.7. Benign Proliferative Breast Diseases

PNI has also been found in non-cutaneous breast lesions, including ductal hyperplasia or ductal carcinoma in situ, lobular hyperplasia, sclerosing adenosis, and papilloma of the breast [125,126,127,128,129,130,131]. The first evidence of PNI in proliferative breast diseases was presented in 1957 by Dr. Lauren Ackerman [132]. Taylor and Norris examined 1000 consecutive breast biopsies at the Armed Forces Institute of Pathology and found 20 cases of PNI [131]. Seventeen of the twenty cases were followed up after ten or more years and found no evidence of adverse outcomes. Gobbi et al. examined 10,000 consecutive breast biopsies at Vanderbilt University Medical Center and found 14 cases of PNI associated with proliferative breast disease [129]. They described that there was no effect on clinical outcomes due to the demonstrated PNI. Elfituri and Emmadi described a 33-year-old woman who underwent a lumpectomy for a suspicious mass. It was found to be a benign proliferative breast disease, including ductal hyperplasia, with evidence of PNI. Follow-up at 20 months showed no evidence of recurrence or malignancy [127]. Therefore, it is important for a pathologist to know that benign proliferative breast diseases are able to demonstrate PNI in order to prevent misdiagnosis and overly aggressive treatment.

### 6.8. Adenomas of the Parotid Gland

**Figure 4:**
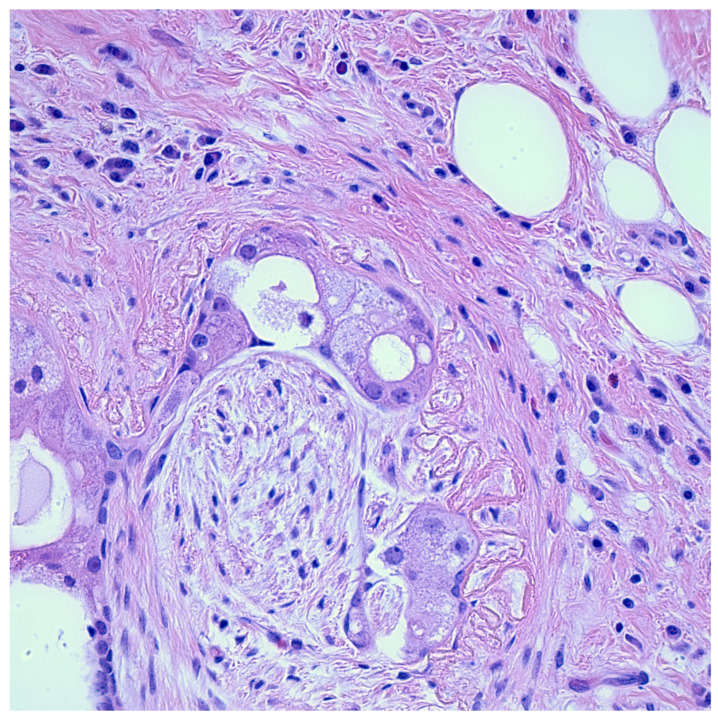
Microscopic image shows a focus on perineural entrapment in a sclerosing polycystic adenoma of the parotid gland. Image was taken at 400× magnification.

After a review of the literature, two reports were found of pleomorphic adenomas of the parotid gland containing PNI. Selesnik and Burt described five head and neck malignant neoplasms and one benign parotid gland adenoma demonstrating PNI [133]. Roncati and Maiorana described a case of a 30-year-old woman with a recurrent pleomorphic adenoma of the parotid gland with PNI [134]. A report by our group also described a case of a 73-year-old woman with a left parotid gland mass, which—upon examination—was diagnosed as a sclerosing polycystic adenoma with evidence of perineural entrapment [135] (Figure 4).

### 6.9. Chronic Pancreatitis

PNI by pancreatic tissue is typically diagnostic of adenocarcinoma, but pathologists must be aware that, in rare cases, chronic pancreatitis can cause PNI [136]. In 1977, Costa et al. found four cases of PNI in pancreatic cells from 304 autopsies [137]. Two cases of benign pancreatic epithelial cells invading the perineural space were reported by Goldman et al. [138]. No signs of malignancy were present in any of the cases. Due to the high prevalence of PNI in pancreatic adenocarcinoma, Moghimi et al. recommended the use of the term “perineural pseudoinvasion” for benign lesions, such as chronic pancreatitis, to prevent confusion with malignant lesions [139].

### 6.10. Gallbladder and Extrahepatic Bile Duct Hyperplasia

After a review of the literature, the earliest report of PNI of gallbladder hyperplasia was by Albores-Saavedra and Henson in 1995 [140]. Three cases were discussed and had been initially misdiagnosed as adenocarcinoma, in part due to the PNI. Albores-Saavedra and Henson also reported four cases of gallbladder florid pyloric gland metaplasia with PNI [141]. All four patients were alive and well 1–7 years post-laparoscopic cholecystectomy. Albores-Saavedra et al. described nine cases of adenomyomatous hyperplasia of the gallbladder with PNI, with five of the cases initially misdiagnosed as adenocarcinoma [142]. That being said, it is important for pathologists to be aware of the possibility of gallbladder and extrahepatic bile duct hyperplasia invading the perineural space to prevent misdiagnosis of adenocarcinoma.

### 6.11. Endometriosis

Several cases have been found with endometriosis demonstrating PNI. Upon review of the literature, the earliest report of endometriosis demonstrating PNI was reported by Roth in 1973 [143]. Lenz et al. described a study of 40 cases of endometriosis and found 5 cases of PNI. It was noted that PNI was found with neural hypertrophy, hyperplasia, and autonomic nervous system involvement in every case [144]. In addition, more research has been conducted on endometriosis and how the pathology may relate to pain symptoms. Liang et al. discussed 64 cases of endometriosis; 38 cases demonstrated PNI [145]. It was found that endometriosis cases with PNI reported overall increased chronic pelvic pain and dysmenorrhea. The authors discussed how the increased trauma and inflammation of the invaded nerves likely caused the increased pain symptoms.

### 6.12. Vasitis Nodosa

Vasitis nodosa is a rare benign proliferation of the epithelium of the vas deferens and causes nodular thickening, most commonly occurring after a vasectomy [146]. Jacobs et al. reported a case of a 36-year-old man post-vasectomy presenting with vasitis nodosa that demonstrated PNI [147]. Balogh and Travis examined 50 cases of vasitis nodosa and found 8 cases of benign PNI [148]. So, it is important for pathologists to recognize the capability of vasitis nodosa to invade the perineural space.

## 7. Histologic Mimics of Perineural Invasion

### 7.1. Peritumoral Fibrosis

Peritumoral fibrosis (PF), a histologic mimic of PNI, is marked by tumor cells that may be encompassing or encompassed by circular arrangements of fibrous tissue [31]. The fibrous tissue can resemble nerve fibers, introducing complications when distinguishing between PF and PNI [149]. Excision of large margins and administration of specific stains, for instance, S-100 protein, are beneficial when distinguishing PF and PNI [149,150]. Compared to H&E staining, S-100 protein IHC staining increases the accuracy of PNI detection [31]. Correctly identifying PF can minimize unnecessary treatments like Mohs surgery and radiation therapy [149,150]. However, PF is a sensitive marker of PNI [150]. Whenever PF is present, the presence of PNI must be carefully determined.

### 7.2. Epithelial Sheath Neuroma

Epithelial sheath neuroma (ESN) is a rare, benign cutaneal tumor of the upper to mid back [31,149]. This histologic finding consists of dermal nerves encapsulated by squamous epithelium [151]. In the encompassing epithelium, lymphocytes and mucin can be noted [149]. ESN itself is not associated with malignancy; however, the histopathology can mimic SCC PNI [149]. Large excision margins should be taken to discriminate between the two pathologies [150].

### 7.3. Re-Excision Perineural Invasion

The insertion of benign squamous epithelial cells into the perineural spaces of dermal nerve fibers is referred to as re-excision PNI (RPI) [149]. The mechanism of RPI development has yet to be fully understood. Some pathologists believe that RPI is caused by wound repair following biopsy, during which reactive eccrine ducts reproduce abnormally into the perineural space [31,149]. Another proposed method of RPI pathogenesis is the incidental introduction of eccrine gland epithelium into the perineural space during a procedure [31,149,152]. Differentiating between RPI and PNI can be difficult, especially when the PNI malignancy is a carcinoma [153]. In cases of specific tumors, for instance, microcystic adnexal carcinoma (MAC), PNI displays a strikingly similar histology to RPI [149]. Scant atypia of the nucleus in the perineural epithelium can be observed in both PNI and PNI associated with MAC [149].

### 7.4. Reactive Neuroepithelial Aggregates (RNEA) of the Skin

As described and proposed by Chen in 2003, reactive neuroepithelial aggregates (RNEA) of the skin are a collection of nerves wrapped in squamous epithelium [154]. The RNEA in Chen’s 2003 case series resembled PNI caused by a carcinoma [154]. According to the study, RNEA was displayed in two samples post-surgical excision and three samples with no associated excision history [154]. The three cases not associated with injury or biopsy illustrated RNEA distribution consistent with eccrine ducts, while one re-excision RNEA case was physically located within an eccrine duct [153,154].

### 7.5. Reparative Perineural Proliferation or Reparative Perineural Hyperplasia

As proposed by Beer in 2009, reparative perineural hyperplasia (RPH) is also a possible differential diagnosis for neoplastic PNI. In his report, Beer presented 10 cases of perineural hyperplasia that manifested post-biopsy [155]. Commonly associated with trauma, RPH is regarded as a reactive process of the perineurium in healing wounds [153,155]. Microscopically, RPH is a proliferation of bland-appearing perineural cells in a concentric arrangement [155]. As with other histologic mimics of malignant PNI, IHC is a critical diagnostic tool. In RPH, perineural cells may be positive for epithelial membrane antigen (EMA) and negative for S-100 protein stain [149,155].

### 7.6. Renaut Bodies

Renaut bodies are structures found in the sub-perineural space of nerves with a propensity to manifest at sites of nerve compression [156]. These EMA-positive entities are long structures of the endoneurium consisting of perineurial cells, fibroblasts, and collagen without obvious nuclear atypia [155,157]. Histologic characteristics that differentiate Renaut bodies from PNI include a lack of nuclear atypia, well-defined borders, and lower amounts of inflammatory infiltrates [158].

## 8. Conclusions

In conclusion, PNI is an essential modality of tumor spread and is linked to poor outcomes in various malignant neoplasms. Nevertheless, it can be seen in benign pathologic conditions as well. Several signaling pathways and neurotrophic factors have been implicated in the development and progression of PNI. Moreover, several in vitro, ex vivo, and in vivo models were developed to enhance our understanding of PNI. We suggest that modulation of these known signaling pathways and neurotrophic factors could be the basis for potential targeted therapy options in tumors where PNI is detrimental to tumor progression.

## Figures and Tables

**Table 1:** Experimental models of perineural invasion in cancer.

Type	Description	Benefits	Limitations
**In Vitro Models**			
**Transwell Model** [35]	Two-dimensional culture using Transwell inserts, cancer cells placed in the apical chamber, chemotactic mechanisms studied	Straightforward, reproducible and cost-effective	Limited in reproducing the full range of cellular behavior observed in the extracellular domain
**Dorsal Root Ganglia Co-Culture Model** [35]	Three-dimensional culture with insertion of dorsal root ganglia in Matrigel, cancer cells placed nearby, and invasion observed via fluorescence	Enhanced visualization and cost-effective	Limited representation of extracellular behavior and does not capture the full range of intercellular interactions between neurons and cancer cells
**Ex Vivo Models**			
**Explanted Vagus Nerve Model** [35]	Harvested vagus nerve placed in the chamber and then on culture media, cancer cells inserted, and invasion observed in culture medium	Valuable for determining the invasive potential of cancer cells of interest	Limited representation of neural microenvironment and lacks the full range of neural-cancer cell interactions
**Explanted Sciatic Nerve Model** [35]	Cancer cells propagated, sciatic nerves were placed on cultured cells, and sciatic nerves were removed for histologic analysis	Allows for investigation of the impact of tumor suppressors on PNI interactions between Schwann and cancer cells	Limited ability to replicate in vivo innervation
**Organoid Model** [35]	Co-culture of dorsal root ganglia with representative organoids	Allows for investigation of intercellular signaling, accurately replicates neural microenvironment	Current technology limits the extent to which intercellular signaling can be observed
**In vivo Models**			
**Heterotopic Xenograft Model** [35]	Cancer cells are directly inserted into the target tissue or native organs, and PNI frequency is analyzed histologically	Diverse set of cell lines able to be studied, and the degree of PNI is readily quantifiable	Limited concurrent neural alterations may not fully mimic human PNI
**Genetically Engineered Mouse Models**	Genetically manipulated mice	Accurate replication of cancer as seen in humans	Restricted neuroplasticity and lack of significant neural invasion
**CAM-Dorsal Root Ganglia Model** [35]	Grafted dorsal root ganglia in chicken embryo chorionic epithelium; cancer cells grafted near neural cells	Mimics neural microenvironment and allows for investigation of roles of molecules or signaling pathways	Restricted observation period due to embryologic immune activation
